# Oxytocin is lower in African American men with diabetes and associates with psycho-social and metabolic health factors

**DOI:** 10.1371/journal.pone.0190301

**Published:** 2018-01-04

**Authors:** Yuval Eisenberg, Lara R. Dugas, Arfana Akbar, Bharathi Reddivari, Brian T. Layden, Elena Barengolts

**Affiliations:** 1 Division of Endocrinology, Diabetes, and Metabolism, Department of Medicine, University of Illinois at Chicago, Chicago, IL, United States of America; 2 Department of Public Health Sciences, Loyola University, Maywood, IL, United States of America; 3 Section of Endocrinology, Department of Medicine, Jesse Brown VA Medical Center, Chicago, IL, United States of America; University of Missouri Columbia, UNITED STATES

## Abstract

**Objective:**

Recently, it has been suggested that oxytocin (OT) has a role in metabolism and neuropsychiatry health and disease, and therefore, it may represent a potential therapeutic target. The current study aimed to investigate relationships between OT and glycemic status along with psycho-social and behavioral factors.

**Design and methods:**

A total of 92 obese or overweight, African American, male subjects were enrolled in the study. Biometric and biochemical data were collected including oral glucose tolerance testing and urinary OT (measured by ELISA). Subjects also completed questionnaires on social and lifestyle factors.

**Results:**

OT levels were found to be significantly lower in subjects with type 2 diabetes mellitus (T2DM) compared to normal glucose tolerance (p<0.05). When stratified by OT tertiles, subjects with higher OT had lower weight, body mass index (BMI) and hemoglobin A1c, but higher eGFR which remained significant after BMI adjustment. The highest OT tertile also had more smokers and more users of psychiatric medications. A stepwise ordered logistic regression supported these findings and could account for 21% of the variation in OT categories (pseudoR^2^ = 0.21).

**Conclusions:**

In this unique population, OT was found lower in subjects with diabetes but higher with better renal function, cigarette smoking and use of psychiatric medications. Future studies are needed to confirm these findings and examine the potential therapeutic role of OT.

## Introduction

The increasing obesity prevalence and associated type 2 diabetes mellitus (T2DM) in the United States means that more than two-thirds of adults over the age of 20 are obese or overweight and nearly 30 million people have diabetes[1]. Minorities have been disproportionately affected including in the African American population[1]. The impact of this health crisis has resulted in a need for new understandings and novel therapies for treating obesity and diabetes.

Multiple peptides produced in the central nervous system contribute to whole body energy homeostasis. Oxytocin (OT) is a neurohypophyseal nonapeptide synthesized primarily in the supraoptic (SON) and paraventricular (PVN) nuclei of the hypothalamus, and its role has been well established in postpartum lactation as well as uterine contracture during labor[2]. Although these have been OT’s traditional functions, recent literature supports a role of OT in energy homeostasis. Animal data has indicated a critical metabolic role for OT including in controlling weight, glucose and lipid metabolism, and even motivation for food consumption and physical activity. Murine models of OT and OT receptor deficiency have increased food intake, develop obesity and reduce energy expenditure[3,4]. Moreover, OT mRNA deficiency has been correlated with severity of hyperphagic obesity and improves with OT peptide administration[5,6]. Taken together, a majority of the mouse models of obesity and T2DM have shown lower circulating OT measures[4,7].

The socio-behavioral impact of OT is another area of active investigation resulting from numerous observed phenomena including mother-child bonding, orgasm, and positive social interaction[8,9]. OT derangements are also described in a variety of disease states that generally effect social interaction including schizophrenia, autism, depression and ADHD[10,11]. Additionally, OT administration for the treatment of some of these diseases has shown some promise including substance abuse such as alcohol, marijuana and tobacco[12,13]. Socio-behavioral factors also play a role in obesity and T2DM, thus, studying these interactions of OT with metabolic and socio-behavioral parameters is needed.

Few studies have been published investigating how OT levels are influenced in obese subjects and/or subjects with the metabolic syndrome or diabetes. Thus far, results between studies are discrepant, some showing higher and others observing lower OT levels in obesity/diabetes[14–16]. Notably, little is known whether OT itself may play a role in pro-social interaction and/or in behaviors that may negatively impact health. In the current study, we explored how urinary OT levels in an understudied, minority patient population (African American male veterans) compared between those with normal glucose tolerance and those with diagnosed T2DM, and whether OT values were associated with biochemical, pro-social behaviors or diet/substance intake.

## Subjects and methods

The study was approved by the Jesse Brown VA Medical Center (JBVAMC) Institutional Review Board. This work was supported in part by a non-profit West Side Institute for Science & Education (WISE), JBVAMC and an NIH grant number UL1RR029879.

### Subjects

African American (AA) male veterans were recruited for a cross-sectional study and completed a single study visit involving biometric and biochemical measures. Samples were collected as part of the Glucose tolerance and vitamin D in African American Male veterans (GluDAAM) cohort study which aimed to evaluate glucose metabolism/biomarker interaction in AA men[17].

The intended subject populations were those with T2DM compared to normal control; fifty subjects in each group. Inclusion criteria were AA men, age 35–70, BMI 22–39.9 kg/m^2^ with 25OH vitamin D level <30 ng/ml. Normal control group had HbA1c values <5.7% and T2DM group were initially defined by HbA1c 6.5–7.4%. Subjects with diabetes could participate so long as they were non-medically treated (lifestyle modification) or were on metformin alone. Exclusion criteria were chronic kidney disease (stages 3b, 4, and 5), chronic glucocorticoid use (3mo or longer), taking non-metformin anti-hyperglycemics, or history of significant health conditions requiring recent (within 6 months) hospitalization.

### Biometrics

All subjects had weight, height, waist circumference, hip circumference and blood pressure measured according to standardized techniques at the day of the study visit[17]. Body mass index (BMI, kg/m^2^) was calculated as weight per height squared (kilograms per square meter) and waist-to-hip-ratio (WHR) was calculated in centimeters as ratio of waist circumference to hip circumference.

### Biochemical measurements

Subjects presented on the study day at 8am after an overnight fast. Baseline laboratory measures were withdrawn from an antecubital vein at time zero, including glucose and insulin (t = 0). Glucose and insulin was then drawn at time points 10, 30, 60, 90, 120 min after consumption of a 75g glucose drink (Glucola). Oral glucose insulin sensitivity (OGIS) based on modeling provided online http://webmet.pd.cnr.it/ogis/ in ml*min^−1^*m^−2^ and Insulin Sensitivity Index (ISI) was calculated based on formula 10^4^ /Square Root of [(fasting glucose × fasting insulin) × (mean glucose × mean insulin)][18,19].

Blood samples were used for measuring HbA1C, 25OHD, glucose, insulin, c-peptide, creatinine, AST, ALT, insulin-like growth factor 1 (IGF-1), total cortisol, total testosterone, C-reactive protein CRP) and leptin at both the JBVAMC clinical laboratory and University of Chicago research laboratory applying laboratory standards of care and references. The analytical methods included ion-exchange high performance liquid chromatography (TOSOH G8 analyzer) for HbA1C and spectrophotometric assay (Siemens Vista 1500 Chemistry analyzer) for glucose, chemiluminescent immunoassay (Siemens ADVIA Centaur XP Chemistry analyzer) for insulin, immunochemiluminometric assay (ICMA, DiaSorin LIAISON analyzer) for 25OHD, and chemiluminescent immunoassay (Siemens Immulite 2000 analyzer) for C-peptide, testosterone, CRP and IGF-1. Lipid measures of total cholesterol (TC), triglycerides (TG), low-density lipoprotein (LDL), and high-density lipoprotein (HDL) were measured by automated standard laboratory methods at the JBVAMC laboratory, and estimated glomerular filtration rate (eGFR) was calculated using the Modification of Diet in Renal Disease (MDRD) equation.

A clean catch urine sample was collected for the measurement of OT and was frozen at -20°C. Samples were sent to the University of Wisconsin-Madison’s Wisconsin National Primate Research Center (WNPRC) for measurement. To measure OT levels, urine samples were thawed and the OT extracted with solid phase extraction (SPE) columns, which purified the urine and removed possible contaminates[20]. The samples were assayed with Assay Design ELISA kits (Enzo Life Sciences, Ann Arbor, MI) per kit instructions as described in Seltzer et al[20]. To compensate for subjects’ potential daily variable fluid intake, creatinine levels were measured in each urine sample and divided into the hormonal concentration ([OT]/[creatinine]), therefore urinary OT levels are expressed as the OT to creatinine ratio (pg/mg creatinine). The creatinine assay procedures are described in detail, see Ziegler et al[21].

### Surveys and Medical history

On the study day, subjects were asked to complete questionnaires on several social and lifestyle factors, as previously described[17]. Subjects reported on level of education completed (primary school, high school, 2-year college, 4-year college, or post-baccalaureate school), employment status (yes or no), yearly income (≤$15,000, $15–40,000, >$40,000), smoking status (yes or no), number of years smoking, and exercise (yes or no). They were also asked questions about consumption of wine, coffee or tea, (yes or no, and quantified per week), as well as active substance abuse status (yes or no). For each subject, medical, psychiatric and medication histories were obtained from chart review and medications were confirmed with patients on the study day.

### Statistical methods

All statistical analyses were performed using STATA (V.13) statistical software (STATA Corp., College Station, TX). Variables were visually inspected prior to analysis, using kernel density testing, and all non-normally distributed data were logarithmically transformed. An alpha p-value of p <0.05 was used to denote statistical significance. Data are presented as means ± standard deviations. To explore associations between OT (independent) and continuous variables, Anova with Bonferroni correction and multiple linear regression analysis with adjustment for confounders were used, while χ^2^ and logistic regression, (odds ratio, OR), were used for categorical analyses. To explore the associations of different levels of OT with specific outcomes, we created a categorical variable, corresponding to the tertiles of OT. T2DM was re-defined after completion of biochemical measures, as HbA1c >6.5 or blood glucose >200 at 2hr time interval of oral glucose tolerance test (OGTT) if not already diagnosed as having diabetes on metformin therapy. To explore the relationship between tertiles of OT and biometric or biochemical factors, or pro-social behaviors and diet/substance intake factors, we performed a stepwise multiple logistic regression analysis and Brant Test of Parallel Regression Assumption post-test.

## Results

### Biometric and biochemical results of study subjects by oxytocin category

Ninety two subjects participated and were stratified by OT category (Table 1). Overall, those subjects with higher urinary OT values had lower body weight (p = 0.035) and consequently BMI (p = 0.025). Glycemic measures of HbA1c, fasting c-peptide and OGTT measured area-under-curve (AUC) insulin and AUC c-peptide were significantly lower in subjects with higher OT values. Additional biochemical measures found IGF-1 to be lower and eGFR elevated with higher measured OT, which remained significant even after adjusting for BMI (p = 0.011, p = 0.047 respectively). At the extremes of OT values (lowest vs. highest category of OT), similar results were observed.

**Table 1:** Characteristics of male veterans by the tertiles of oxytocin level.

Characteristic	1^st^ (N = 31)	2^nd^ (N = 31)	3^rd^ (N = 30)	Over-all p-value	Adjusted p-value (BMI)
OXT, pg/mg Cr	2.9 ± 0.9	5.8 ± 1.1	14.9 ± 7.8**		
Oxytocin-log	0.44 ± 0.14	0.76 ± 0.06	1.15 ± 0.17**		
**General**					
Age, years	56.4 ± 6.6	57.0 ± 7.2	56.6 ± 4.4	0.875	0.557
Weight, kg	100.9 ± 18.4	95.4 ± 19.6	90.5 ± 19.0**	**0.035**	-
BMI, kg/m^2^	33.0 ± 4.9	30.3 ± 4.2	29.8 ± 4.8*	**0.025**	-
WHR	1.01 ± 0.09	0.97 ± 0.06	0.97 ± 0.09	0.062	0.804
**Glycemic**					
A1C, %	6.3 ± 0.9	6.0 ± 0.8	5.8 ± 0.8*	**0.021**	0.388
FPG, mg/dL	113.9 ± 22.6	105.5 ± 20.3	108.0 ± 28.6	0.335	0.516
Insulin-F, mIU/L	22.1 ± 15.9	15.1 ± 11.6	20.9 ± 42.6	0.861	0.262
C-peptide-F, pmol/L	987 ± 571	592 ± 452	564 ± 691	**0.006**	0.145
OGIS	360 ± 85	380 ± 114	406 ± 102	0.083	0.895
AUC glucose	29088 ± 9734	26112 ± 9110	25759 ± 9176	0.175	0.726
AUC insulin	15679 ± 12690	11866 ± 6747	9159 ± 6459**	**0.006**	0.070
AUC C-peptide	451119 ± 209493	356978 ± 171442	287891 ± 233594**	**0.001**	**0.013**
**Hormones**					
Leptin, ng/mL	25.2 ± 19.9	17.6 ± 13.1	18.0 ± 17.6	0.102	0.963
IGF-1, ng/mL	140 ± 44	137 ± 51	112 ± 42*	**0.020**	**0.011**
CRP, mg/L	5.72 ± 5.17	6.77 ± 6.48	4.77 ± 4.05	0.597	0.091
**Chemistry**					
ALT/AST	1.68 ± 0.69	1.58 ± 0.55	1.27 ± 0.47**	**0.008**	0.059
Creatinine, mg/dL	1.1 ± 0.2	1.1 ± 0.1	1.0 ± 0.2	0.068	0.091
eGFR, mL/min/1.73 m^2^	84.0 ± 15.9	90.0 ± 15.4	94.4 ± 23.7*	**0.032**	**0.047**
TG mg/dL	139.9 ± 84.8	105.2 ± 34.7	135.4 ± 108.0	0.215	0.505
LDL mg/dL	95.0 ± 36.7	91.2 ± 28.1	86.6 ± 29.0	0.303	0.344
**Social, N [%]**					
Education, college	10 [32.3]	11 [35.5]	7 [23.3]	0.873	0.883
Smoking	7 [23.3]	13 [41.9]	16 [53.3]*	**0.020**	**0.029**
Smoking, years	6.3 ± 10.2	13.6 ± 13.9	19.2 ± 14.1**	**0.017**	**0.028**
Exercise	12 [40.0]	17 [54.8]	12 [40.0]	1.000	0.841
Wine	9 [36.0]	7 [29.2]	2 [11.1]	0.082	0.339
Coffee/Tea, >1drink/day	16[51.6]	19 [61.3]	23 [76.7]	0.045	0.109
Employment	9 [45.0]	6 [35.3]	2 [14.3]	0.073	0.066
Income <$15,000	9 [34.6]	16 [53.3]	17 [65.4]	0.068	0.131
**Medical history, N [%]**					
HTN	18 [58.1]	23 [74.2]	22 [73.3]	0.308	**0.034**
DM2	23 [74.2]	16 [51.6]	12 [40.0]*	**0.004**	0.098
Metformin	14 [45.5]	10 [32.3]	6 [20.0]*	**0.039**	0.477
PTSD	5 [16.1]	7 [22.6]	9 [31.6]	0.470	0.537
Depression	8 [24.8]	10 [32.3]	12 [40.0]	0.497	0.342
Opioid misuse	11 [35.5]	12 [38.7)	16 [53.3]	0.325	0.363
Psych conditions, any	16 [51.6]	21 [67.7]	21 [70.0]	0.248	0.518
Psych meds, any	3 [9.7]	9 [30.0]	11 [36.7]*	**0.018**	**0.047**
N of all meds	7.5 ± 5.8	9.2 ± 6.7	10.4 ± 8.2	0.098	**0.042**
N of psych conditions	1.0 ± 0.2	1.3 ± 1.2	1.4 ± 1.2	0.157	0.373

Abbreviations: A1C, hemoglobin A1C; AUC, area under the curve; CRP, C reactive protein; FPG, fasting plasma glucose; IGF, insulin-like growth factor; F, fasting; N, number; OGIS, oral glucose insulin sensitivity (Mari index); Psych, psychiatric; WHR, waist to hip ratio. Data are Mean SD or N [%]. For categorical variables data are number [%] for “yes” answer. Bolded values represent p<0.05.

*p<0.05 and

**p<0.001 for 3^rd^ vs. 1^st^ tertile.

### Psycho-social results of study subjects by oxytocin category

Significantly more smokers were found in the highest category of OT (53% vs 42% vs. 23%, overall p = 0.02) which remained significant after adjusting for BMI. Male veterans with the highest OT levels had between a 3 to 5-fold increase in the odds of being current smokers (OR 3.80, CI 1.2–11.4), drinking tea and coffee (OR 3.10, CI 1.01–9.26) and currently using psychiatric medications (5.40, CI 1.3–21.9) respectively, after controlling for BMI (Table 2). Subjects with highest OT levels were approximately 80% less likely to have T2DM (OR: 0.2, p = 0.004), drink wine (OR: 0.22, p = 0.08), currently be employed (OR: 0.2, p = 0.073), but this lost significance when adjusted for BMI. Although there was no difference in psychiatric conditions based on OT levels, including specifically PTSD and depression, the use of psychiatric medications was higher with higher OT values and remained significant after BMI adjustment.

**Table 2:** Odds ratio (OR) for the highest vs. the lowest (reference) oxytocin level tertiles in male veterans.

Characteristics*	DM2	Smoking	Coffee/tea	Wine	Psych Meds	Employed
OR	0.20	3.80	3.10	0.22	5.40	0.20
95% CI	0.07–0.60	1.2–11.4	1.02–9.26	0.04–1.19	1.3–21.9	0.04–1.16
p value	0.004	0.019	0.045	0.080	0.018	0.073
Adjusted p-value	0.098	**0.029**	0.109	0.339	**0.044**	0.066

*Characteristics of lifestyle (smoking, drinking tea, coffee, wine) and employment were assessed by a questionnaire. Adjusted p-value, adjusted for BMI.

### Oxytocin results by glycemic status

**Fig 1:**
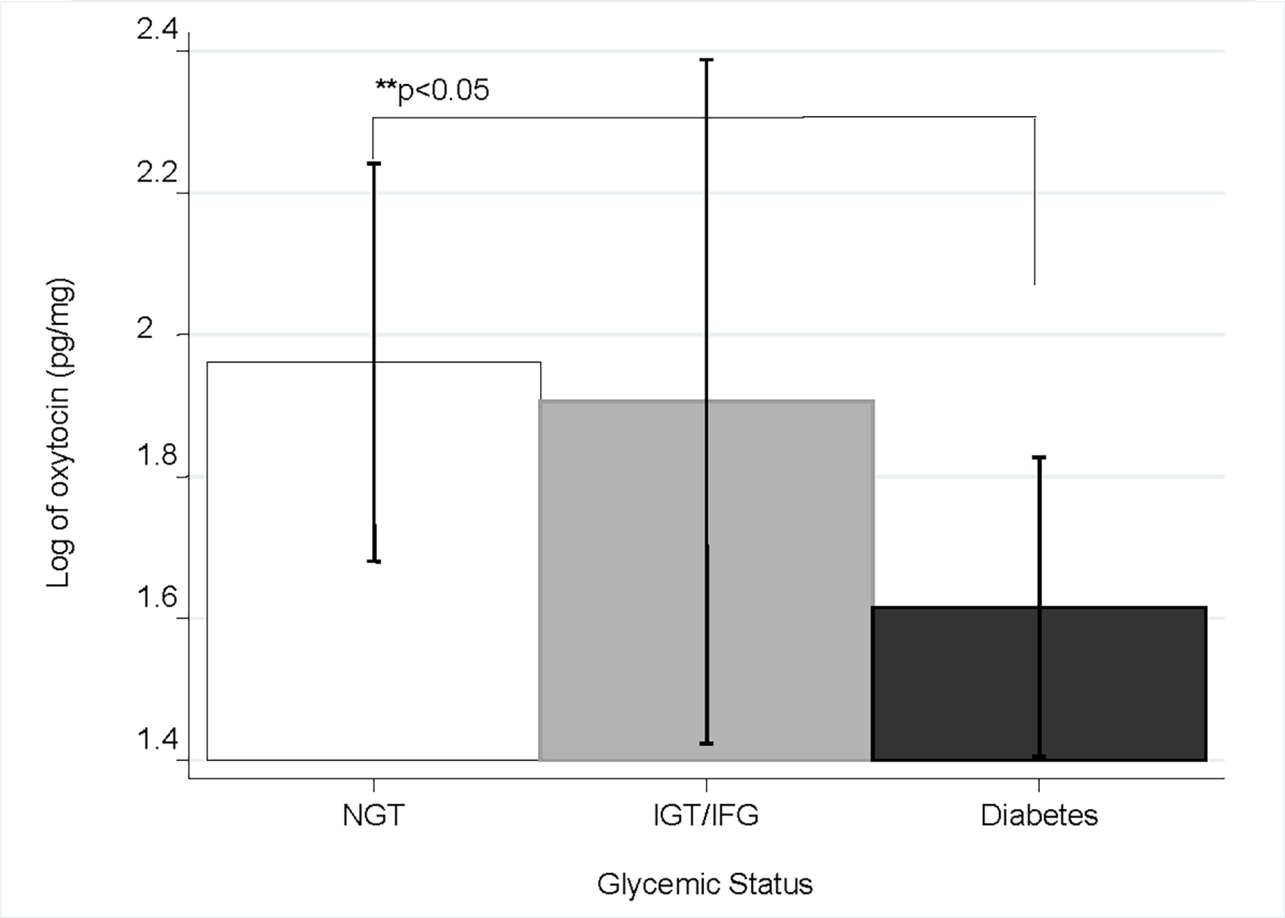
Oxytocin values by glycemic status. NGT = Normal glucose tolerance, IGT = Impaired glucose tolerance, IFG = Impaired fasting glucose.

After completion of the OGTT, subjects were classified as either normal glucose tolerance (NGT), impaired fasting glucose (IFG), impaired glucose tolerance (IGT) and having diabetes (T2DM) according to ADA recommendations[22] (Fig 1). Subjects with T2DM had significantly lower OT values than those with normal glucose tolerance (1.62 ± 0.74 vs 1.96 ± 0.79, p<0.05). There was no difference, however, between either NGT or T2DM to those with IGT or IFG.

### OT regression analyses

Amongst the entire study population, there was a positive correlation between OT values and kidney function, as indicated by higher eGFR which remained significant when accounting for BMI (correlation coefficient 0.232, p = 0.026). Stepwise linear regression showed an independent relationship between eGFR and OT (regression coefficient = 0.009, standard error = 0.004, 95% CI (0.0004–0.0169), p = 0.041).

We next examined the relationship between OT levels (dependent) and metabolic and social parameters (Table 3). In a stepwise ordered logistic regression, hypertensives had 2.4-fold increase in the odds of being in a higher category compared to veterans without hypertension (OR 2.42, CI 0.84–6.95) but the difference did not reach significance. On the other hand, smokers (OR 4.09, CI 1.53–10.90) and veterans using psychiatric medications (4.98, CI 1.50–16.54) had greater than a four-fold increase in the odds of being in a higher category OT. Finally, eGFR and fasting insulin were positively associated with being in a higher category of OT, while increasing values for the natural log of AUC C-peptide was associated with being in the lower category of OT. The final model explains 21% of the variance between OT categories amongst study subjects (Table 3).

**Table 3:** Stepwise ordered logistic regression analysis* with oxytocin tertiles as the dependent variable.

Characteristics*	Odds Ratio	95% CI	P-value
Hypertension (Y/N)	2.42	0.84 to 6.95	0.100
Natural log AUC C-Peptide	0.24	0.12 to 0.50	0.001
Fasting insulin	1.03	1.00 to 1.05	0.016
Glomerular filtration rate	1.04	1.01 to 1.07	0.014
Smoking (Y/N)	4.09	1.53 to 10.90	0.005
Psychiatric medications (Y/N)	4.98	1.50 to 16.54	0.009

*Only variables that had a p <0.15 were included in the final model. Model R^2^ = 0.21. Odds Ratio (OR) compares highest to lowest tertile of oxyocin. The following independent variables were considered for the model: waist circumference, fasting insulin, natural log AUC C-Peptide, creatinine, eGFR, IGF-1, diabetes status (Yes/No), smoking status (Yes/No), psychiatric Meds (Yes/No), total number of medications (any). Post-test estimation: Brant test of parallel regression assumption indicated that the parallel regression had not been violated.

Abbreviations: AUC, area under the curve.

## Discussion

Subjects in the present study represent a unique population of AA male veterans for whom no data on OT levels exist in the literature. This population has a disproportionately high rate of obesity and dysglycemia, as well as high rates of comorbid psychiatric disease. Few studies have assessed for differences in OT between groups with metabolic syndrome or T2DM, especially in human subjects. Notably, there have also been discordant results between the published data. Qian and colleagues showed lower serum OT levels in T2DM patients which correlated with a variety of metabolic markers (BMI, waist-to-hip ratio, HbA1c, HOMA-IR)[16]. The association remained significant when regression modeling was utilized for 2hr plasma glucose, BMI, total cholesterol as well as T2DM. A group of mostly female subjects with the metabolic syndrome also showed similar results[15]. Lower OT levels were seen in those with prediabetes or T2DM (n = 89, OT = 1222.46+/- 514.55 pg/mL) compared to control normo-glycemic subjects (n = 69, OT = 2323.42+/- 848.68 pg/mL, p<0.001). In fact, OT correlated negatively with HbA1c and positively with fasting plasma glucose[15]. Discrepant results, however, were seen when OT values were assessed in a cohort of older men with the metabolic syndrome[14]. Sera from 540 men from the MINOS cohort (a prospective study of osteoporosis in men age 50–85) were assayed for OT and found to be higher in those with metabolic syndrome. When adjusted for confounders (including biometrics- age, height, leptin, 25OHD, osteocalcin, testosterone and social aspects-current smoking, coffee drinking, alcohol use, physical activity), higher OT (> = 0.74 pg/mL) predicted metabolic syndrome (OR: 2.06 (1.33–3.18), p<0.005)[14].

Differences in the described data may stem from several sources. The MINOS cohort and the current study consisted only of men, while the other studies had majority women. Furthermore, the time of OT assay from sample collection varied widely between studies. The samples for the current study were frozen and processed within 6–12 months while in the MINOS study samples were collected and stored 20 years prior to assay. In the MINOS cohort, nearly 25% of their OT values fell below the level of detection (<0.4pg/ml, n = 133, total = 540) for their RIA. The degree of dysglycemia also varied between studies, some studies having up to 50% T2DM total[16] while others had only a small percentage of T2DM subjects (7% of total)[14]. Additionally, the duration of diabetes/dysglycemia in these populations varied and may affect circulating OT levels. The raw OT levels may have also been influenced by the omission of the extraction processing step leading to reported values several orders of magnitude higher than the others[15].

In the current study cohort, subjects with highest levels of OT had lower HbA1c, weight and BMI. When stratified by OT tertiles, those with T2DM were significantly more likely to be found in the lowest range of OT. There were not, however correlation or independent association between OT and lipid measures, as was seen in other studies[16]. During exploratory statistical modeling, several other dysglycemia-related factors were found associated with higher OT tertiles. For example, higher values of AUC c-peptide were associated with being more likely in the lower tertiles of OT; however, fasting insulin levels has a discordant relationship. AUC c-peptide has been described as a superior measure of beta-cell health, with higher values indicating higher beta-cell stress[23]. Overall, these data suggest a favorable metabolic effect of higher OT levels.

While only a few studies assessing OT levels in human subjects have been published, several studies have attempted intranasal OT administration for weight loss or treatment of metabolic syndrome (based on animal models showing promising anorexogenic effects). OT seems to reduce reward driven food intake behavior[24], and in fact reductions in ad libitum caloric intake was shown after OT administration[25]. Significant weight loss was even achieved with 8 weeks of intranasal OT therapy. A recent review describes these results in more detail[26].

In the current population, IGF-1 levels declined with higher OT levels and appeared independent of age and BMI. These results are consistent with previous *in vivo* animal data suggesting physiological link between IGF-1 and OT. Administration of IGF-1 within the SON in rats acutely inhibits the activity of OXT neurons[27]. Additionally, circulating OT has been shown to be higher in older compared to younger mice, possibly explained by the loss of approximately one-third of IGF-1 receptor immunoreactive cells in both SON and PVN in older compared to younger mice[28]. There are, however, reported region specific differences in IGF-1 regulation of OT, with stimulatory effects shown within ovarian tissues[29].

A unique finding from the current study was the correlation between OT values and measures of renal function. The OT measurements performed were on urinary samples, but were corrected for urinary creatinine measures and therefore would not be expected to correlate with serum estimates of eGFR. Specifically, those in the lowest category of OT were found to have significantly lower eGFR. Several studies have shown a potential anti-inflammatory and specifically antioxidative effect of OT. In rats exposed to the cytotoxic effects of cisplatin, OT reduced formation of reactive oxygen species (ROS) as well as histological evidence of nephrotoxicity[30]. Similarly, in rat models of ischemia/reperfusion, OT administration alleviated renal injury via a reduction in oxidative damage from ROS[31]. Additionally, studies in healthy rats and dogs found a significantly increased GFR after both acute and chronic (9 days of infusion) administration of intravenous OT[32]. Further study is necessary to confirm this finding and to determine if OT therapy could have a nephroprotective effect.

A plethora of literature exists on OT and sociality, supporting its role as a hormone involved in bonding, empathy, trust, and social cognition[8,9]. In a variety of psychopathologies (including schizophrenia, schizoaffective disorder and autism), which have difficulties with interpersonal interactions, OT levels have been found lower than controls[11]. Very little is known about OT and social behaviors or demographics in our study population. One recorded behavior, cigarette smoking, showed an interesting relationship with measured OT in the current study. Notably, OT levels were higher in smokers, and the duration of smoking was also higher in the highest category of OT which remained significant even when adjusting for BMI. Animal models of acute nicotine exposure have shown increased brain activity in a variety of regions, including the oxytocinergic PVN and SON[33]. Nicotine induced OT release has been posited as a cause of premature birth in smokers owed to its increased uterine contractile sensitivity[34]. Chronic administration of nicotine appears to upregulate OT receptor binding in regions of the brain involved in stress and emotion regulation, and these neuro-adaptations likely influence nicotine-seeking behaviors[35]. Interestingly, OT administration in rats had positive effects on treating the somatic component of nicotine withdrawal[36]. While human data is quite limited, several small clinical studies utilizing OT for treatment of substance dependence (nicotine, alcohol, cannabis, cocaine) have shown some promise and intranasal OT is under active investigation for smoking cessation (NCT02595749)[13].

An additional factor influencing OT in the current cohort was the use of psychiatric medications. There was a high burden of psychiatric disease that was not different between OT categories, but medication use was significantly higher among those in the in the highest OT group. As described previously, lower OT levels correlated with a variety psychiatric conditions[10,37]. Studies involving medication-free subjects show specifically more impulsiveness and negative emotionality[37]. The influence of psychiatric medications on oxytocinergic systems is not well understood, however, medication-related improved psychological health outcome might result in OT changes. In our study, specific medications were not recorded, nor was measure of response to therapies which might have provided insight into the OT differences that were observed.

Controversy exists within the literature regarding methodologies of OT measures. OT has been measured in urine[20,21,38], serum/plasma[14–16] and saliva[39] with some discrepancies even with the same chosen body fluid in similar populations[14,16]. Type of assay (radioimmunoassay vs enzyme-linked immunoassay) as well as specific detection antibody vary between studies. Even sample processing (e.g. sample extraction) has been debated[40]. The current study utilized, what the authors believe to be, the best currently available practice, which seems to favor solid phase sample extraction (as recommended by most manufacturers of commercially available assays), and the most widely used kit from Enzo Life Sciences.

There are limitations to our study. Although some between group differences were found, causality cannot be inferred in this cross-sectional study. Both the size and heterogeneity of the population selected may have influenced the ability to detect any notable further differences. Similarly, while subjects reported cigarette and substance use, the time from last use was not documented and could influence the individual measure. And finally, single measures of urinary OT may not be reflective of an individual’s overall OT levels.

## Conclusion

In this population of overweight or obese AA male veterans, urinary OT levels were associated with measures of glycemic status with higher values favoring a healthier metabolic phenotype. Those in the lowest category of OT had significantly higher HbA1c and reduced eGFR and being in the lowest OT group was associated with T2DM. Several interesting associations with OT were observed in this cohort for both behavioral (smoking), psychological (use of psychiatric medications) and biochemical measures (eGFR and IGF-1). Future studies are necessary to determine the magnitude and impact of OT derangements in subjects with T2DM and metabolic syndrome. Additionally, future studies are needed to investigate a potential positive impact of OT on kidney function, psychometabolic health and smoking cessation.

## Supporting information

S1 File Eisenberg data—11.10.2017.xlsx. Complete supplementary data file.
(XLSX)
